# Effect of a Hexagonal Barbell on the Mechanical Demand of Deadlift Performance

**DOI:** 10.3390/sports5040082

**Published:** 2017-10-24

**Authors:** Jason Lake, Freddie Duncan, Matt Jackson, David Naworynsky

**Affiliations:** Department of Sport and Exercise Sciences, University of Chichester, College Lane, 19 6PE Chichester, UK; freddie_duncan@hotmail.co.uk (F.D.); mattjackson10@hotmail.co.uk (M.J.); dnaworynsky@gmail.com (D.N.)

**Keywords:** strength and conditioning, linear position transducer, biomechanical analysis, resistance exercise

## Abstract

This study compared typical mechanical variables of interest obtained directly from barbell motion during deadlift performance with a conventional (CBD) and a hexagonal barbell (HBD). Eleven men, proficient with both deadlift variations, volunteered to participate in the study (age: 20.3 ± 0.6 years; height: 175.5 ± 8.5 m; mass: 88.7 ± 19.0 kg; CBD 1RM: 183 ± 22 kg; HBD 1RM: 194 ± 20 kg). During the first session, CBD and HBD 1RM was assessed; during the second session, they performed 3 sets of 1 CBD repetition with 90% 1RM; and in session three, they repeated this process with the HBD. Barbell displacement was recorded at 1000 Hz and mechanical parameters derived from this. Significantly heavier loads were lifted during HBD (6%, *p* = 0.003). There were no significant differences between barbell displacement (*p* = 0.216). However, HBD was performed significantly faster (15%, *p* = 0.012), HBD load was accelerated for significantly longer (36%, *p* = 0.004), and significantly larger mean forces underpinned this (6%, *p* < 0.001), with more work having been performed (7%, *p* < 0.001) at greater power outputs (28%, *p* < 0.001). The results of this study showed that heavier HBD loads can be lifted through the same range of motion faster, and that this load is accelerated for significantly longer. The strategies used to achieve these differences could have a significant effect on training outcomes.

## 1. Introduction

The deadlift is an exercise that is often included in athlete strength and conditioning programs [1,2,3]. Traditionally the deadlift has been performed using a conventional barbell (conventional barbell deadlift: CBD) and the main deadlift variations have come in the form of the technique used to perform the lift, with lifters applying a conventional (hands gripping the barbell outside of the legs) or sumo (hands gripping the barbell between the legs) [2], in addition to different loading strategies [3,4]. The introduction of the hexagonal barbell provided one of the most common deadlift variations used by strength and conditioning coaches for their athletes; however there is a paucity of research on the effect its use has on the mechanical demands of the deadlift [1,3] (hexagonal barbell deadlift: HBD). Researchers have shown that significantly higher peak force, velocity and power can be achieved with the HBD [1,3]. Furthermore, researchers have shown that the vastus lateralis makes a significantly greater contribution during the HBD. The biceps femoris makes a significantly greater contribution during the concentric phase of CBD, while the erector spinae makes a significantly greater contribution to the eccentric phase of the CBD [1]. However, some researchers found no significant difference between CBD and HBD peak force [5].

One of the potential limitations of these studies is that they used the combined method, combining force plate derived force data with motion capture system or linear position transducer derived barbell velocity. This method has been shown to significantly over estimate power during lower-body resistance exercises [6,7]. Furthermore, because of the incompatibility between the typical conventional and hexagonal barbell lengths and most force plate dimensions, obtaining measures of work and power directly from the barbell may be more methodologically appropriate [6,8,9]. This is because the force plate method relies on the assumption that barbell and body system mass do not change during performance. If, as is the case as the barbell leaves the ground during deadlift performance, they do then this assumption is violated and methodological integrity is compromised. 

It should also be noted that recent research has questioned the use of peak instantaneous metrics like peak force and peak power [10,11]. While these metrics are popular and can be useful, they represent a very small part of the movement of interest. For example, if data are recorded at 1000 Hz peak instantaneous values will only represent 1 millisecond. Therefore, it has been recommended that researchers and practitioners consider variables like mean force, the work performed and power achieved during whole phases of motion to get a fuller understanding of the mechanical demands of resistance exercises. 

Therefore, there is currently a need to explore other mechanical variables of interest using appropriate methodology to contribute to the small but growing body of research. Doing so will help strength and conditioning practitioners make more informed decisions about the potential suitability and benefits of the CBD and HBD. Investigators have often quantified the mechanical demand of resistance exercises by studying the peak instantaneous values of variables like force, velocity and power [3,12]. However, it has been suggested that mechanical demand should be quantified using data averaged over phases of interest, because this provides greater insight into the demand placed on the individual over the phase of interest, rather than the demand placed on the individual over what are typically very short sampling durations [12,13]. Therefore, mean force, mean velocity, and mean power provide insight about how hard individuals must push or pull an implement to move it, and the effect this has on the implement, respectively. While studying barbell motion and deriving measures, like force, work, and power does not provide a comprehensive picture of the mechanical demand that is placed on the lifter, it provides researchers with a foundation from which they can decide whether the area requires further research. Therefore, the aim of this study was to compare the mechanical demands of the CBD and HBD. It was hypothesised that the HBD would enable participants to lift heavier loads. Additionally, it was hypothesised that the differences in hand placement (the HBD has handles outside the legs with the palms facing one another either side of the lower-leg) would result in significantly greater barbell displacement during the HBD, and this, combined with the heavier loads, would make significantly greater mechanical demands on participants. 

## 2. Materials and Methods

### 2.1. Subjects

Eleven healthy men who were proficient with both deadlift variations volunteered to participate in the study (age: 20.3 ± 0.6 years; height: 176.2 ± 8.4 m; mass: 88.5 ± 18.2 kg; CBD 1RM: 183 ± 22 kg; HBD 1RM: 194 ± 20 kg). After experimental aims, procedures and risks were explained, participants completed a health history questionnaire and written informed consent, and the study was approved by the institutional ethical review board. 

### 2.2. Procedures

Participants attended three separate laboratory-based sessions that were separated by a minimum of 48 h to enable sufficient recovery [3,14,15]. The laboratory sessions included 1RM testing (both exercises, counterbalanced) and two experimental sessions where CBD and HBD techniques were examined. Before data collection, a pilot study was conducted to ensure that all study procedures worked efficiently. 

Before each testing session, participants performed a standardised warm up to optimise lifting performance. Participants completed a 5-min cycle at 60 revolutions per minute and at 100 W on a Wattbike cycle ergometer (Wattbike Pro, Wattbike Ltd., Nottingham, UK) followed by a 5-min period of dynamic stretching, targeting the muscle groups used during both deadlift techniques (see Lake et al. [11]). Then participants performed a series of CBD (session two) and HBD (session three) warm up sets with submaximal loads (ranging from 40 to 85% 1RM) for 2–3 repetitions.

### 2.3. One Repetition Maximum Testing

**Figure 1:**
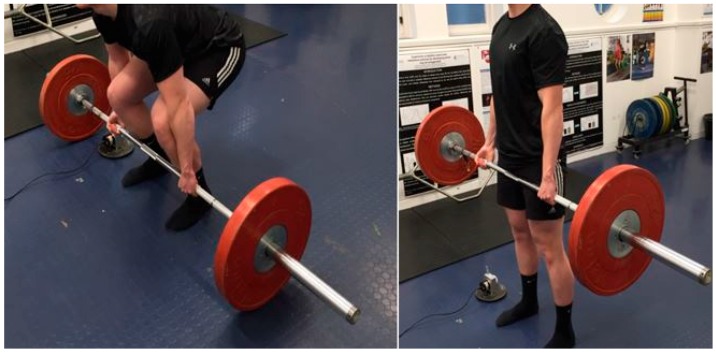
Demonstration of the conventional deadlift technique, from the beginning (**left**) to the end of the concentric phase of the lift (**right**).

**Figure 2:**
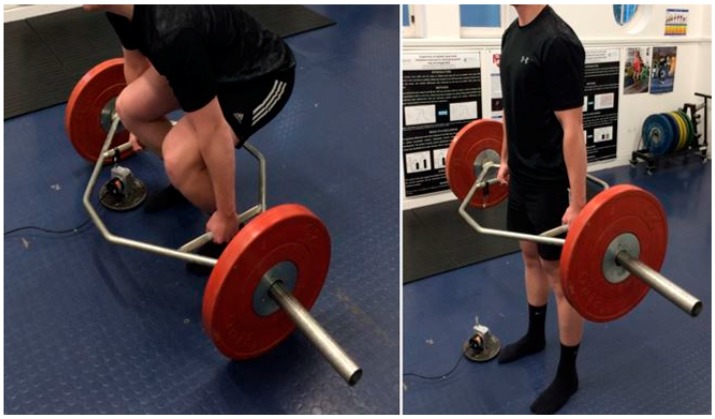
Demonstration of the hexagonal barbell deadlift technique, from the beginning (**left**) to the end of the concentric phase of the lift (**right**).

In session one, CBD (using a York Olympic Barbell, York Barbell, UK, Daventry, UK) and HBD (using a hex barbell, Pullum Sports, Luton, UK) 1RM was assessed. Exercise order was counter-balanced. The barbells were loaded with a load equal to the participant’s body mass (Nelco Bumper Plates, Nelco Ltd., Mumbai, India) to begin with and this load was increased following the guidelines presented by Haff and Triplett [16] (Figure 17.1, page 453). As participants had sufficient deadlifting experience (assessed by visual inspection of a qualified coach), each participant had knowledge of their estimated 1RM based on recent training logs. Two to four minutes were provided between maximum attempts to allow participants to recover [3]. The maximum load lifted for one repetition classified as a legal CBD (barbell not lowered at any point during the ascent and upon completion of the movement, knees locked in a fully extended position and shoulders retracted [3]), was recorded as the 1RM. To minimize the influence of fatigue on performance, a 30-min recovery period was allocated between the CBD and HBD 1RM tests. A reverse grip was used during the CBD (Figure 1), while a parallel (palms facing) overhand grip was used during the HBD (Figure 2) and all deadlifting was performed bare footed. Participants were permitted to use chalk to aid grip.

### 2.4. Sub-Maximal Testing

In the second session, participants performed the CBD, lifting a load of 90% CBD 1RM. In powerlifting competition, lifters are given three attempts to maximize the amount of weight they can lift, and so participants performed three single lift repetitions in total (3 × 1 repetition) with a minimum of 2 min rest between each, with more rest taken if participants felt it was necessary to produce maximal performance [2]. In session three, participants repeated the process performed during the second session with the HBD.

### 2.5. Data Acquisition and Processing

Barbell displacement was recorded during all tests for the entire repetition at 1000 Hz using a linear position transducer (Chronojump Boscosystem, Barcelona, Spain) attached to each barbell, near the participant’s hand. Raw data was exported from Chronojump Software (version 1.6.2) and analysed in Microsoft Excel (Microsoft, Seattle, WA, USA). The dependent variables were the mean velocity of the barbell during the lifting phase, total barbell displacement, phase duration, percentage of the lifting phase the barbell was accelerated for, mean force applied to the barbell during the lifting phase, the work performed on the barbell during the lifting phase, and the power achieved during the lifting phase. The lifting phase was identified as the period between the first positive velocity to peak displacement (the deadlift finish position) [3]. Velocity was obtained by differentiating the displacement signal that had been filtered using a Butterworth filter with a cut-off frequency of 10 Hz (chosen based on results of residual analysis). Mean velocity was obtained by averaging velocity over the lifting phase. The percentage of the lifting phase that each barbell was accelerated for was calculated by calculating the time to peak velocity and dividing this by total lifting phase duration. Mean force was obtained by first numerically differentiating velocity-time data, applying a Butterworth filter with a cut-off frequency of 10 Hz (chosen based on results of residual analysis), and then performing the following calculation: Barbell force = (Barbell mass × Barbell acceleration) + (Barbell mass × *g*) where *g* is the acceleration of gravity [6,8]; this force was then averaged over the lifting phase. Power was calculated by multiplying the velocity of the barbell by the force applied to the barbell, and mean power was obtained by averaging the barbell power over the lifting phase. Work was obtained by calculating the area under the power-time curve. The reliability of all dependent variables was assessed using the intraclass correlation coefficient, which showed that all dependent variables were highly reliable with ICC R values of between 0.80 and 0.98 [17].

### 2.6. Statistical Analysis

Skewness and Kurtosis ratios indicated that all dependent variables were normally distributed. Assessment was conducted by dividing the statistic value by the standard error value; any value with a standardized skewness and kurtosis score inside the ±2 cut-off points were classified as normally distributed [18]. Paired sample *t* tests were used to assess differences between dependent variables obtained from the CBD and HBD condition. Effect sizes (ES) were used to quantify the practical relevance of differences between dependent variables obtained from the CBD and HBD, and were categorised using the scale presented by Hopkins et al. [19] where 0.20, 0.60, 1.20, 2.0, and 4.0 represented small, moderate, large, very large and extremely large effects. With the exception of the effect sizes that were calculated in Microsoft Excel (Microsoft, Seattle, WA, USA), all statistical analysis was performed SPSS v.23 (SPSS Inc., Armonk, NY, USA) was used to perform all statistical analysis. A significance level was set at *p* < 0.05 and all data was presented as mean ± SD. 

## 3. Results

Participants were able to lift significantly greater loads during the HBD (194 ± 20 vs. 183 ± 22 kg; *p* = 0.003, ES = 0.503). The results of the comparison between the variables used to describe the mechanical demand of the CBD and HBD are presented in Table 1. While the mean velocity of the barbell was significantly faster during HBD (15%, *p* = 0.012, ES = 0.459), the total displacements of the two barbells were not significantly (1%, *p* = 0.216) or practically (ES = 0.126) different. However, CBD duration was significantly longer than the HBD equivalent (20%, *p* = 0.012, ES = 0.923). Furthermore, load was accelerated for significantly longer during the HBD (36%, *p* = 0.004, ES = 1.778). Finally, mean force (6%, *p* < 0.001, ES = 0.494), work (7%, *p* < 0.001, ES = 0.512) and mean power (28%, *p* < 0.001, ES = 0.889) was significantly greater during HBD.

## 4. Discussion

The aim of this study was to test the hypothesis that the HBD enables the use of heavier loads and in so doing places a greater mechanical demand on the lifter. This was done by obtaining mechanical parameters directly from barbell motion and averaging them over the lifting phase. In general, our results supported this hypothesis. Significantly greater loads were lifted over a similar range of motion. Because the HBD was heavier, their displacement required significantly greater mean force to be applied to the Hex bar, meaning that significantly more work was performed at a significantly faster rate during the HBD. 

To date, three studies have studied differences between the CBD and HBD [1,3,5]. However, only one of these has compared differences between their 1RM [1]. The results of the current study showed that participants were able to lift significantly more (Table 1) during the HBD. This is contrary to the study that compared CBD and HBD and found no significant differences (181 ± 27 kg vs. 181 ± 28 kg) [1]. The 1RM values presented by Swinton et al. showed that HBD 1RM was 8% larger than the CBD 1RM. Interestingly, the 1RM data presented by Swinton et al. [3] and in the present study suggest that the improved leverages afforded by the Hex barbell facilitate the lifting of greater loads. Furthermore, the results of the current study show that these heavier loads are lifted through the same range of motion. 

Although the total displacement of the barbell during the CBD and HBD was not significantly different, the duration of the lifting phase was significantly longer during the CBD. This resulted in a significantly faster mean velocity during the HBD, which agrees with related data (peak velocity) presented by Camara et al. [1] and Swinton et al. [3]. Interestingly, Camara et al. [1] studied CBD and HBD with 65% and 85% 1RM, while Swinton et al. [3] used loads ranging from 10% to 80% 1RM. Therefore, the current study is the first to study HBD with 90% 1RM and it shows that in spite of this heavier load, the Hex barbell is still displaced significantly faster. This may be explained by the fact that the barbell was accelerated for significantly longer during the CBD and because of the significantly smaller horizontal displacements that have been presented in the literature [3]. The significantly faster velocity and longer time spent accelerating the barbell could make the HBD an excellent alternative resistance exercise for strength and conditioning coaches looking for a resistance exercise to maximize these qualities. 

The significantly greater loads that were lifted during the HBD required a significantly larger mean force to displace them. This is to be expected, because ultimately the displacement of a mass is dependent on a combination of how much force is applied to it across the duration of the lifting phase (impulse) [10]. While this finding suggests that the HBD may provide an excellent alternative resistance exercise for strength and conditioning coaches looking for a resistance exercise to maximize the force application, mean barbell velocity, in addition to how long the barbell is accelerated for, previous research must be considered. For example, Swinton et al. [3] showed that the HBD was positioned significantly closer to the athlete (based on horizontal displacement from the start to end position). Furthermore, Camara et al. [1] showed that the way the main muscles that contribute to deadlift performance are recruited changes when the Hex barbell is used. For example, they showed that the vastus lateralis makes a significantly greater contribution throughout the HBD, while the biceps femoris makes a significantly greater contribution during the concentric phase of CBD. Therefore, while the HBD may maximise some elements of mechanical output during deadlift performance, the strategy used to achieve these changes. This could have important implications for training outcomes. However, more detailed biomechanical analysis and training study based research is needed to corroborate this. 

There were significant differences between the work performed displacing the barbell during CBD and HBD performance. Furthermore, because HBD lifting phase duration was significantly shorter, there were significant differences in the mean power achieved during CBD and HBD deadlift performance. Work performed during HBD has not been studied before and it would appear that the significant differences in the load that was lifted and therefore mean force, had a significant impact on this variable. Furthermore, because the effect that the Hex barbell had on phase duration was moderate to large the mean power achieved by displacing the larger HBD loads at a faster velocity was significantly larger. Not only does this finding agree with previous research [1,3], it also suggests that, in addition to mean power been a more appropriate variable to quantify this capacity [10], it is also sensitive enough to reflect differences that have been found in the inappropriate peak power alternative that has been used. Whether this means that mean power should be used over peak power is beyond the scope of this paper and readers are encouraged to read the recent review by Winter et al. [10] to help inform their decision.

Although this study provides useful information that contributes to the growing body of research, it is not without its limitations. Given the amount of research that has been done into the most appropriate resistance exercise power, many may feel that the method used in this study was inappropriate. However, we would argue that, while that argument would be warranted if we’d studied resistance exercises where all of the lifter and barbell system load was on the force plate at the beginning, this isn’t the case during the deadlift. Comfort et al. [20] overcame this limitation when studying power clean variations by instructing participants to start the movement from a position where the barbell was held off of ground. However, for obvious reasons, this was not possible during deadlift performance with 90% 1RM. Therefore, we feel confident that the linear position transducer method was appropriate for this study. Another limitation of this study could be found in our use of the linear position transducer. For example, Swinton et al. [3] have demonstrated that load influences horizontal displacement during the HBD. One of the main limitations of the linear position transducer approach is that, with the exception of some commercially available systems, it cannot differentiate between horizontal and vertical displacement. Consequently, using a linear position transducer to record barbell velocity data may have resulted in some bias in the results because it relies on assumption that any horizontal barbell displacement is consistent across loads, and this may not be the case. Finally, the authors are cognisant that the mechanical parameters examined in the present study do not necessarily reflect muscle work or any differences in the movement strategies that might be employed to displace these two distinctly different barbells. However, in light of the results of this and other studies [1,3,5] more detailed biomechanical analysis could be warranted to quantify these. 

To summarise, the results of this study showed that significantly heavier loads can be lifted during HBD. Furthermore, they are moved through the same range of motion significantly faster, and the load is accelerated for significantly longer. These differences extend to the work performed and the mean power achieved in displacing the different barbells. Strength and conditioning practitioners should bear in mind that the strategies used to achieve these differences could have a significant effect on training outcomes.

## Figures and Tables

**Table 1:** The mean (SD) results of the comparison between the variables used to describe the mechanical demand of the CBD and HBD.

Mechanical Demands	CBD	HBD
Total displacement (m)	0.50 (0.05)	0.50 (0.04)
Mean velocity (m/s)	0.29 (0.10)	0.33 (0.09) *
Duration (s)	1.89 (0.56) *	1.50 (0.28)
Acceleration (% of duration)	60 (14)	82 (11) *
Mean force (N)	1613.3 (194.8)	1705.6 (179.1) *
Work (J)	803.5 (110.6)	859.2 (107.1) *
Mean power (W)	459.9 (154.9)	589.3 (136.1) *

* Significantly different (*p* < 0.05); CBD = conventional barbell deadlift; HBD = hexagonal barbell deadlift.
